# Severe Brain Atrophy Predicts Poor Clinical Outcome After Endovascular Treatment of Acute Basilar Artery Occlusion: An Automated Volumetric Analysis of a Nationwide Registry

**DOI:** 10.3389/fnagi.2021.720061

**Published:** 2021-08-17

**Authors:** Chang Liu, Hansheng Liu, Deping Wu, Zhiming Zhou, WenGuo Huang, Zhilin Wu, Wenjie Zi, Qingwu Yang

**Affiliations:** ^1^Department of Neurology, Xinqiao Hospital and The Second Affiliated Hospital, Army Medical University (Third Military Medical University), Chongqing, China; ^2^Department of Neurology, Yijishan Hospital of Wannan Medical College, Wuhu, China; ^3^Department of Neurology, Chinese Medical Hospital of Maoming, Maoming, China; ^4^Department of Neurology, Yunfu People's Hospital, Yunfu, China

**Keywords:** brain atrophy, acute basilar artery occlusion, computed tomography, endovascular treatment, automatical analysis

## Abstract

**Background:** Brain atrophy globally reflects the effects of preexisting risk factors and biological aging on brain structures and normally predicts poor outcomes in anterior circulation stroke. However, comparing with these patients, acute basilar artery occlusion (ABAO) impairs infratentorial regions frequently and might benefit from brain atrophy due to the resulting residual space to reduce tissue compression and thus improve prognosis, which raises doubts that current understandings for prognostic roles of brain atrophy are also applicable for ABAO. Therefore, this study aims to evaluate brain atrophy automatically from CT images and investigates its impact on outcomes of ABAO following endovascular treatment (EVT).

**Methods:** A total of 231 ABAO who underwent EVT from the BASILAR registry were enrolled. Brain atrophy was quantified as the ratio of brain parenchymal volume to cerebrospinal fluid volume on baseline CT. The primary outcome was the modified Rankin Scale (mRS) score at 3 months.

**Results:** The frequency of favorable outcomes (90-day mRS ≤ 3) was significantly lower in the severe atrophy group (*P* = 0.014). Adjusted logistic models revealed that severe brain atrophy was significantly negatively associated with favorable outcome incidence (*P* = 0.006), with no relationship with either in-hospital or 90-day overall mortality (all *P* > 0.05). Adding a severe atrophy index into the baseline model obviously enhanced its discriminatory ability in predicting the outcome by obviously increasing areas under the receiver operating characteristic curve, net reclassification improvement algorithm, and integrated discrimination improvement algorithm values (all *P* < 0.05).

**Conclusion:** Severe brain atrophy did not improve in-hospital or overall mortality but impaired the long-term recovery after EVT. This objective and automated marker has the potential to be incorporated into decision-support methods for treating ABAO.

## Introduction

Acute basilar artery occlusion (ABAO) accounts for nearly 1% of all forms of ischemic stroke (Mattle et al., 2011). It is a devastating neurological disorder with high mortality that leaves a substantial part of survivors severely disabled (Mattle et al., 2011). Although endovascular treatment (EVT) has been increasingly applied as a common strategy in daily clinical practice for patients with ABAO, the latest BASICS randomized controlled trial focusing on the management of ABAO failed to demonstrate statistically the benefit of this intervention over medical management therapy under current EVT indications (Langezaal et al., 2021). To further ameliorate the outcomes of patients with ABAO treated with EVT, novel key prognostic markers are urgently needed to improve clinical decision-making systems and to identify patients suitable for EVT.

Brain atrophy, indicating the loss of brain cells or their connections, has recently been presented as a new, reliable imaging marker for predicting poor functional outcomes in patients with anterior circulation stroke treated with intravenous thrombolysis or EVT (Lauksio et al., 2020; Pedraza et al., 2020). However, compared with anterior circulation stroke, which damages cerebral hemispheres, in patients with ABAO, the subtentorial structures are frequently impaired; such patients might benefit from brain atrophy, which provides compensation space and increases tolerance of space-occupying conditions, thereby decreasing the probability of brain herniation, decreasing mortality, and promoting recovery (Mattle et al., 2011; Delcourt et al., 2020). The markedly different infarction locations between ABAO and anterior circulation stroke have raised doubts that the current understanding of the prognostic roles of brain atrophy is applicable to ABAO. However, due to the relatively low incidence of ABAO, the association between brain atrophy and outcomes of these patients with ABAO after EVT has not been explored to the best of our knowledge.

Previous methods of evaluating brain atrophy have mainly relied on the subjective visual experience of the neurologists performing the evaluation, which might limit the use of brain atrophy for outcome evaluation in clinical practice (Appleton et al., 2020). Nevertheless, a recently developed automated volumetric algorithm named CTseg has enabled the rapid and objective estimation of the degree of global brain atrophy after mapping to the standard brain map and quantifying brain parenchymal volume (BPV) and cerebrospinal fluid volume (CFV) by CT, which is the most frequent type of brain image used to diagnose ischemic stroke (Adduru et al., 2020; Brudfors et al., 2020). Therefore, based on our previous multicenter BASILAR registry and the CTseg automatic algorithm, this study aims to explore the role of brain atrophy in determining clinical outcomes among patients with ABAO treated with EVT.

## Materials and Methods

### Study Design and Participants

The BASILAR study was a nationwide, prospective registry of EVT plus medical management vs. medical management alone for patients who were confirmed with an acute symptomatic and radiological ABAO from 47 comprehensive stroke centers in China between January 2014 and May 2019. The details of the study protocol have been previously published (Writing Group for the BASILAR Group et al., 2020).

In the present analysis, we evaluated the degree of atrophy automatically from CT slices to further explore the impact of brain atrophy on clinical outcomes among patients with ABAO after EVT (Adduru et al., 2020; Brudfors et al., 2020). We included consecutive patients in the BASILAR registry who met the following criteria: (1) treated with EVT, (2) accepted non-contrast-enhanced CT (NECT) scanning before the endovascular intervention, and (3) NECT slice thickness ≤ 5 mm (to guarantee the accuracy of the analysis). From 829 patients in the BASILAR registry, a total of 231 patients treated with EVT were enrolled in this study. In addition, another 66 patients with ABAO who had been treated with medical management alone (NECT thickness also ≤5 mm) were further enrolled to verify whether brain atrophy data can improve clinical decision-making.

### Standard Protocol Approvals, Registrations, and Patient Consents

The BASILAR was registered on the Chinese Clinical Trial Registry (ChiCTR1800014759). This study was approved by the research board at each participating center, and informed consent was obtained from all patients or their authorized representatives.

### Procedures and Data Collection

In addition to CT images acquired at admission, we retrospectively collected data on baseline characteristics, including age, sex, vascular risk factors (i.e., diabetes mellitus, hypertension, atrial fibrillation, and hyperlipidemia), National Institute of Health Stroke Scale (NIHSS) at admission, and posterior circulation-Alberta Stroke Program Early scores (PC-ASPECTS). Collateral circulation status was assessed by the posterior circulation collateral score (PC-CS) based on the presence of potential collateral pathways on CT angiography (van der Hoeven et al., 2016). Successful reperfusion was defined as a modified thrombolysis-in-cerebral-infarction (mTICI) score higher than 2a at the end of the intervention. An independent core imaging laboratory, blinded to clinical outcomes, assessed all digital subtraction angiographies and imaging data.

Primary functional outcomes at follow-up were assessed using the 90-day modified Rankin Scale (mRS). A score on the 90-day mRS of 4–6 was defined as a poor outcome. sICH was defined as a newly observed intracranial hemorrhage leading to an increase of four points on the NIHSS before worsening or an increase of two points in one category (Liu et al., 2020; Writing Group for the BASILAR Group et al., 2020). Early neurological improvement (ENI) was defined as a reduction of the NIHSS score from the baseline score of >8, or a return to 0, 24 h after EVT (Guenego et al., 2021).

### Brain Atrophy Degree Evaluation

We estimated brain atrophy automatically based on the CTseg algorithm (https://github.com/WCHN/CTseg), developed by the Ashburner group at the Wellcome Trust Centre for Neuroimaging (Brudfors et al., 2020). This routine spatially normalized brain CT images in the standard brain space at the Montreal Neurological Institute by flexible Bayesian modeling and further segmented the total BPV and CFV after skull stripping. Compared with previous methods, CTseg leads to a more robust segmentation that can better handle images with considerable noise and/or large morphological variability (Brudfors et al., 2020). Brain atrophy was then categorized by tertiles in the ratio of BPV–CFV as follows: first tertile (>3.349), mild atrophy; second tertile (3.018–3.349), intermediate atrophy; and third tertile (<3.018), severe atrophy.

### Statistical Analysis

Depending on the normality of the distribution as assessed by the Kolmogorov–Smirnov test, continuous variables were compared using Student's *t*-test for independent samples, or the Mann–Whitney *U*-test or Kruskal–Wallis test for non-normal data. Proportion tests for categorical variables were performed using the chi-square test or Fisher's exact test. The data were presented as mean ± SD, median [interquartile range (IQR)], or as number (percentage), where appropriate. To determine the independent prognostic factors for favorable outcomes, the binary and multivariable logistic regression analyses were performed, and the results were summarized as odds ratios (ORs) with 95% CIs. The restricted cubic spline analyses were employed to characterize the dose–response association and to explore the potential linear or nonlinear relationship between atrophy status and clinical outcome. We used three predefined “knots” (inter-spline dividing values of the independent variable) for transforming the BPV/CFV values for restricted cubic spline analysis (10, 33, and 90th percentiles). The tests for nonlinearity were performed first. If this test was not statistically significant, the test result for overall association and linearity was checked, with a significant result indicating a linear association. We also analyzed the heterogeneity of the effect of brain atrophy status within subgroups based on sex, age (≤65 or >65 years), mTICI (0–2a or 2b−3), NIHSS score (<25 or ≥25), and ASITN/SIR (<2 or ≥2). The areas under the receiver operating characteristic curves (AUCs) were calculated and compared using the DeLong's test. The incremental effects of the severe level of the brain atrophy index for outcome prediction were examined using the net reclassification improvement algorithm (NRI) and the integrated discrimination improvement algorithm (IDI), with the baseline model as a reference. The threshold for statistical significance was set at *P* < 0.05. All statistical analyses were performed using the R software version 3.6.1 (https://www.r-project.org).

## Results

### Baseline Characteristics of the Study Population

The clinical manifestations of the study population at admission are shown in Table 1. The average age was 62.74 years and 74.46% of them were men. A total of 152 patients (65.80%) had a history of hypertension and 53 (22.94%) patients had a history of diabetes mellitus. A total of 79 (34.20%) patients achieved favorable outcomes. The PC-ASPECTS score, the PC-CS, and the proportion of mTICI ≥ 2b were significantly higher in patients with favorable outcomes than in those with poor outcomes (90-day mRS ≤3 vs. >3; all *P* < 0.01). In addition, the baseline NIHSS score, the onset-to-puncture time, the puncture-to-recanalization time, and the onset-to-recanalization were significantly elevated in patients with poor outcomes (all *P* < 0.05).

**Table 1:** Baseline characteristics of the study population.

	**All patients (*N* = 231)**	**mRS ≤ 3 (*N* = 79)**	**mRS > 3 (*N* = 152)**	***P***
Age, years, mean ± SD	62.74 ± 12.18	61.23 ± 13.35	63.52 ± 11.49	0.175
Men, (*n*%)	172 (74.46)	56 (70.89)	116 (76.32)	0.460
Baseline NIHSS, median (IQR)	26.0 (17.0–32.0)	23.0 (11.0–30.0)	29.0 (20.0–34.0)	<0.001
Initial PC-ASPECTS, median (IQR)	8 (7–9)	9 (8–10)	7 (6–8)	<0.001
Admission SBP, mmHg, mean ± SD	144.94 ± 23.42	146.52 ± 23.72	144.12 ± 23.30	0.461
Admission DBP, mmHg, mean ± SD	83.86 ± 15.17	83.38 ± 16.09	84.11 ± 14.71	0.729
24 h NIHSS after EVT, median (IQR)	27.0 (12.0–34.0)	9.0 (3.0–17.5)	32.0 (23.0–35.0)	<0.001
7 d NIHSS after EVT, median(IQR)	19.0 (6.5–35.0)	3.0 (1.0–8.0)	31.5 (18.0–36.0)	<0.001
Pre-onset mRS				
0	206 (89.18)	71 (89.87)	135 (88.82)	0.794
1	20 (8.66)	7 (8.86)	13 (8.55)	
2	5 (2.16)	1 (1.27)	4 (2.63)	
BPV/CFV	3.20 (0.45)	3.30 (0.41)	3.16 (0.47)	0.022
**History of risk factors**, ***n*****(%)**				
Hypertension	152 (65.80)	56 (70.89)	96 (63.16)	0.304
Diabetes mellitus	53 (22.94)	16 (20.25)	37 (24.34)	0.592
Dyslipidemia	73 (31.60)	30 (37.97)	43 (28.29)	0.176
Atrial fibrillation	45 (19.48)	19 (24.05)	26 (17.11)	0.276
Smoking	85 (36.80)	32 (40.51)	53 (34.87)	0.484
TIA	5 (2.16)	1 (1.27)	4 (2.63)	0.841
**TOAST classification**, ***n*****(%)**				0.262
LAA	151 (65.37)	45 (56.96)	106 (69.74)	
CE	58 (25.11)	25 (31.65)	33 (21.71)	
SOE	6 (2.60)	2 (2.53)	4 (2.63)	
SUE	16 (6.93)	7 (8.86)	9 (5.92)	
**Imaging parameters**				
Occlusion site, *n* (%)				0.044
BA distal	76 (32.90)	34 (43.04)	42 (27.63)	
BA middle	70 (30.30)	16 (20.25)	54 (35.53)	
BA proximal	42 (18.18)	13 (16.46)	29 (19.08)	
V4	43 (18.61)	16 (20.25)	27 (17.76)	
PC-CS score, median (IQR)	4 (3–6)	5 (4–6)	4 (3–6)	<0.001
**Treatment procedure**, ***n*****(%)**				
Intravenous thrombolysis	49 (21.21)	15 (18.99)	34 (22.37)	0.330
Anesthesia				0.450
General	83 (35.93)	31 (39.24)	52 (34.21)	
Local	148 (64.07)	48 (60.76)	100 (65.79)	
**Reperfusion status**, ***n*****(%)**
mTICI				
0–2a	42 (18.18)	5 (6.33)	37 (24.34)	0.009
2b−3^a^	189 (81.18)	74 (93.67)	115 (75.66)	
**Treatment delay, median (IQR), min**				
Onset to puncture	324.5 (230.5, 494.2)	266.0 (172.0, 384.0)	354.0 (250.0, 506.0)	0.005
Puncture to recanalization	103.0 (69.5, 144.5)	86.0 (60.0, 120.0)	113.0 (79.0, 167.0)	<0.001
Onset to recanalization	449.0 (326.0, 627.7)	365.0 (279.0, 499.0)	485.0 (365.0, 649.0)	<0.001

a *mTICI score of 2b or 3 indicates satisfied recanalization*.

*BA, basilar artery; mTICI, modified thrombolysis in cerebral infarction; PCA, posterior cerebral artery; V4, V4 segment of vertebral artery; CE, cardioembolism; NIHSS, National Institutes of Health Stroke Scale; PC-ASPECTS, posterior circulation Alberta Stroke Program Early CT Score; SBP, systolic blood pressure; DBP, diastolic blood pressure; SOE, stroke of other determined cause; SUE, stroke of undetermined cause; TIA, transient ischemic attack; TOAST, Trial of ORG 10172 in Acute Stroke Treatment; BPV, brain parenchymal volume; CFV, cerebrospinal fluid volume*.

Patients were stratified into three groups according to their brain atrophy levels (Table 2). The average age in the highest tertile group was obviously elevated compared with the others [lowest tertile (*N* = 77): 58.19 ± 11.15; intermediate tertile (*N* = 77): 60.26 ± 10.45; highest tertile (*N* = 77): 69.75 ± 11.75, *P* < 0.001]. We also detected significant differences in the proportions of men, atrial fibrillation, and dyslipidemia among these three groups (all *P* < 0.05). None of the other risk factors, including systolic blood pressure, diastolic blood pressure, initial PC-ASPECTS score, and baseline NIHSS, were remarkably affected by the degree of atrophy (all *P* > 0.05).

**Table 2:** Baseline clinical characteristics according to brain atrophy levels.

	**Mild atrophy (*N* = 77)^**a**^**	**Intermediate atrophy (*N* = 77)^**a**^**	**Severe atrophy (*N* = 77)^**a**^**	***P***
Age, years, mean ± SD	58.19 ± 11.15	60.26 ± 10.45	69.75 ± 11.75	<0.001
Male, (*n* %)	53 (68.83)	70 (90.91)	49 (63.64)	<0.001
Baseline NIHSS, median (IQR)	26.0 (18.0–32.0)	26.0 (18.0–34.0)	27.0 (16.0–32.0)	0.914
PC-ASPECTS, median (IQR)	8.0 (7.0–9.0)	8.0 (7.0–9.0)	8.0 (6.0–9.0)	0.608
Admission SBP, mmHg, mean ± SD	142.49 ± 26.00	143.90 ± 21.21	148.43 ± 21.98	0.260
Admission DBP, mmHg, mean ± SD	84.00 ± 17.30	83.86 ± 12.57	83.73 ± 15.44	0.994
NIHSS at 24 h after EVT, median (IQR)	23.0 (9.0–35.0)	27.0 (15.0–34.0)	30.0 (13.0–35.0)	0.780
NIHSS at 7 days after EVT, median (IQR)	15.0 (5.0–35.0)	18.0 (9.0–35.0)	23.0 (8.0–35.0)	0.443
Pre-onset mRS (%)				0.267
0	72 (93.51)	64 (83.12)	70 (90.91)	
1	4 (5.19)	11 (14.29)	5 (6.49)	
2	1 (1.30)	2 (2.60)	2 (2.60)	
**History of risk factors**, ***n*****(%)**				
Hypertension	48 (62.34)	52 (67.53)	52 (67.53)	0.735
Diabetes mellitus	24 (31.17)	17 (22.08)	12 (15.58)	0.069
Dyslipidemia	26 (33.77)	36 (46.75)	11 (14.29)	<0.001
Atrial fibrillation	13 (16.88)	9 (11.69)	23 (29.87)	0.013
TIA	2 (2.60)	2 (2.60)	1 (1.30)	0.815
**TOAST classification**, ***n*****(%)**				0.139
LAA	51 (66.23)	58 (75.32)	42 (54.55)	
CE	17 (22.08)	13 (16.88)	28 (36.36)	
SOE	2 (2.60)	2 (2.60)	2 (2.60)	
SUE	7 (9.09)	4 (5.19)	5 (6.49)	
**Imaging parameters**				
Occlusion site, *n* (%)				0.326
BA distal	25 (32.47)	19 (24.68)	32 (41.56)	
BA middle	22 (28.57)	25 (32.47)	23 (29.87)	
BA proximal	13 (16.88)	16 (20.78)	13 (16.88)	
V4	17 (22.08)	17 (22.08)	9 (11.69)	
PC-CS score, median (IQR)	5 (4–6)	4 (2–5)	5 (3.5–6)	0.026
**Treatment procedure**, ***n*****(%)**				
Intravenous thrombolysis	18 (23.38)	16 (20.78)	15 (19.48)	0.834
Anesthesia				0.073
General	30 (38.96)	33 (42.86)	20 (25.97)	
Local	47 (61.04)	44 (57.14)	57 (74.03)	
**Reperfusion status**, ***n*****(%)**				
mTICI				0.542
0–2a	11 (14.29)	15 (19.48)	16 (20.78)	
2b−3^b^	66 (85.71)	62 (80.52)	61 (79.22)	
**Treatment delay, median (IQR), min**				
Onset to puncture	315.0 (240.7, 475.7)	330.0 (191.0, 589.5)	324.0 (250.1, 440.5)	0.977
Puncture to recanalization	102.0 (69.8, 148.0)	107.0 (79.5, 144.8)	99.50 (58.0, 136.5)	0.508
Onset to recanalization	430.0 (345.8, 617.0)	454.0 (315.0, 710.0)	449.5 (331.3, 569.8)	0.836

a *Brain atrophy was categorized by tertile based on the ratio of brain parenchymal volume–cerebrospinal fluid volume (BPV–CFV); mild atrophy (first tertile): BPV–CFV >3.349; intermediate atrophy (second tertile): BPV–CFV 3.018–3.349; severe atrophy (third tertile): BPV–CFV < 3.018*.

b *mTICI score of 2b or 3 indicates complete recanalization*.

*PC-CS, posterior circulation collateral system score; BA, basilar artery; mTICI, modified thrombolysis in cerebral infarction; PCA, posterior cerebral artery; V4, V4 segment of vertebral artery; CE, cardioembolism; NIHSS, National Institutes of Health Stroke Scale; PC-ASPECTS, posterior circulation Alberta Stroke Program Early CT Score; SBP, systolic blood pressure; DBP, diastolic blood pressure; SOE, stroke of other determined cause; SUE, stroke of undetermined cause; TIA, transient ischemic attack; TOAST, Trial of ORG 10172 in Acute Stroke Treatment*.

### The Impact of Brain Atrophy on Clinical Outcome

As shown in Table 3, favorable outcomes were least likely among patients with severe atrophy, with lower percentages than in the other atrophy subgroups (mild atrophy vs. intermediate vs. severe: mRS ≤ 3, 41.56 vs. 37.66 vs. 23.38%, *P* = 0.044; mRS ≤ 2, 40.25 vs. 28.57 vs. 22.08%, *P* = 0.045). In the adjusted analysis, intermediate brain atrophy had the same prognostic values as mild brain atrophy (all *P* > 0.05). However, compared with mild brain atrophy, favorable outcomes were less likely to occur in the severe atrophy group [adjusted OR with 95% CI: mRS ≤ 3, 0.21 (0.07–0.62), *P* = 0.006; mRS ≤ 2, 0.18 (0.05–0.58), *P* = 0.006; mRS ≤ 1, 0.22 (0.06, 0.69), *P* = 0.014; Table 3).

**Table 3:** The impact of brain atrophy on clinical outcome.

**Characteristics**	**Atrophy levels**	**No./No. (%)**	***P*-value**	**OR (95%CI)**	***P***	**Adjusted OR (95%CI)**	***P***
**EFFICACY OUTCOME**							
**90-day outcome**							
mRS, median (IQR)	Mild	5 (1–6)	0.137^a^	Reference	Reference	Reference	Reference
	Intermediate	5 (2–6)		1.23 (0.68, 2.34)^c^	0.493	0.69 (0.34, 1.41)^d^	0.311
	Severe	5 (3–6)		1.96 (1.06, 3.65)^c^	0.033	2.43 (1.17, 5.12)^d^	0.018
mRS 0-3	Mild	32 (41.56)	0.044^b^	Reference	Reference	Reference	Reference
Intermediate	29 (37.66)		0.85 (0.44, 1.62)	0.621	2.11 (0.82, 5.74)	0.131
	Severe	18 (23.38)		0.43 (0.21, 0.85)	0.017	0.21 (0.07, 0.62)	0.006
mRS 0-2	Mild	31 (40.25)	0.045^b^	Reference	Reference	Reference	Reference
	Intermediate	22 (28.57)		0.59 (0.30, 1.16)	0.128	1.13 (0.42, 3.08)	0.814
	Severe	17 (22.08)		0.42 (0.20, 0.84)	0.016	0.18 (0.05, 0.58)	0.006
mRS 0-1	Mild	26 (33.77)	0.066^b^	Reference	Reference	Reference	Reference
	Intermediate	17 (22.08)		0.56 (0.27, 1.13)	0.108	0.93 (0.32, 2.70)	0.890
	Severe	14 (18.18)		0.44 (0.20, 0.91)	0.029	0.22 (0.06, 0.69)	0.014
**NIHSS SCORE**							
Change from baseline at	Mild	0.00 (−4.00–2.00)	0.455^a^	Reference	Reference	Reference	Reference
24 h, median (IQR)	Intermediate	0.00 (−4.00, 2.00)		1.78 (−1.44 to 5.00)^e^	0.278	0.39 (−2.71 to 3.49)^e^	0.805
	Severe	0.00 (2.00, 2.00)		2.48 (−0.74 to 5.70)^e^	0.131	1.54 (−1.68 to 4.77)^f^	0.346
Change from baseline at	Mild	−4.0 (−14.00, 2.00)	0.285^a^	Reference	Reference	Reference	Reference
5–7 d, median (IQR)	Intermediate	−2.00 (−15.00, 2.00)		1.69 (−2.44 to 5.81)^f^	0.421	−0.87 (−4.56 to 2.82)^f^	0.642
	Severe	0.00 (−9.00, 4.00)		3.78 (−0.35 to 7.90)^f^	0.072	2.02 (−1.82 to 5.86)^f^	0.300
ENI^g^	Mild	14 (18.18)	0.916^b^	Reference	Reference	Reference	Reference
	Intermediate	13 (16.88)		0.91 (0.39, 2.11)	0.832	2.50 (0.77, 8.94)	0.139
	Severe	15 (19.48)		1.09 (0.48, 2.46)	0.837	1.99 (0.65, 6.51)	0.227
**SAFETY OUTCOMES**							
Mortality in hospital	Mild	15 (19.48)	0.770^b^	Reference	Reference	Reference	Reference
	Intermediate	15 (19.48)		1.00 (0.45, 2.23)	1.000	0.90 (0.35, 2.34)	0.832
	Severe	12 (15.58)		0.76 (0.33,1.76)	0.526	0.69 (0.22, 2.07)	0.511
Mortality at 90 d	Mild	29 (37.66)	0.502^b^	Reference	Reference	Reference	Reference
	Intermediate	34 (44.16)		1.18 (0.62, 2.25)	0.621	0.59 (0.22, 1.52)	0.285
	Severe	36 (46.75)		1.45 (0.77, 2.78)	0.254	1.26 (0.47, 3.36)	0.636
SICH	Mild	4 (5.19)	0.768^b^	Reference	Reference	Reference	Reference
	Intermediate	3 (3.90)		0.74 (0.14, 3.47)	0.700	0.86 (0.13, 5.57)	0.870
	Severe	5 (6.49)		1.27 (0.32, 5.30)	0.732	0.47 (0.08, 2.83)	0.404

a *Wilcoxon test*.

b *Chi-square test*.

c *Common odds ratio*.

d *Adjusted common odds ratio; adjusted estimates of outcome were calculated using multiple regression, taking the following variables into account: age, sex, dyslipidemia, atrial fibrillation, baseline NIHSS score, baseline PC-ASPECTS, mTICI, PC-CS, and onset to recanalization time*.

e *β-values were estimated from a univariate linear regression model*.

f *β-values were estimated from a multivariable linear regression model; adjusted estimates of outcome were calculated using multiple regression, taking the following variables into account: age, sex, dyslipidemia, atrial fibrillation, baseline NIHSS score, baseline PC-ASPECTS, mTICI, PC-CS, and onset to recanalization time*.

g *ENI: early neurological improvement was estimated by a reduction of > 8 or return to 0 on NIHSS compared with baseline score at 24 h after EVT*.

*EVT, endovascular treatment; NIHSS, National Institutes of Health Stroke Scale; mRS, modified Rankin Scale score at 90 days*.

Previous studies have demonstrated that brain atrophy can promote the survival of patients with anterior circulation stroke (Lee et al., 2010). However, we found no significant correlations between brain atrophy degree and NIHSS score alterations at 24 h, NIHSS score alterations at 5–7 days, or the proportion of ENI (all *P* > 0.05). Both the unadjusted model and the multivariate analysis with adjustment for confounders indicated that brain atrophy level could not improve in-hospital or 90-day mortality (all *P* > 0.05).

### Association of Severe Brain Atrophy With Traditional Risk Factors in Predicting Outcomes of ABAO Treated With EVT

**Figure 1:**
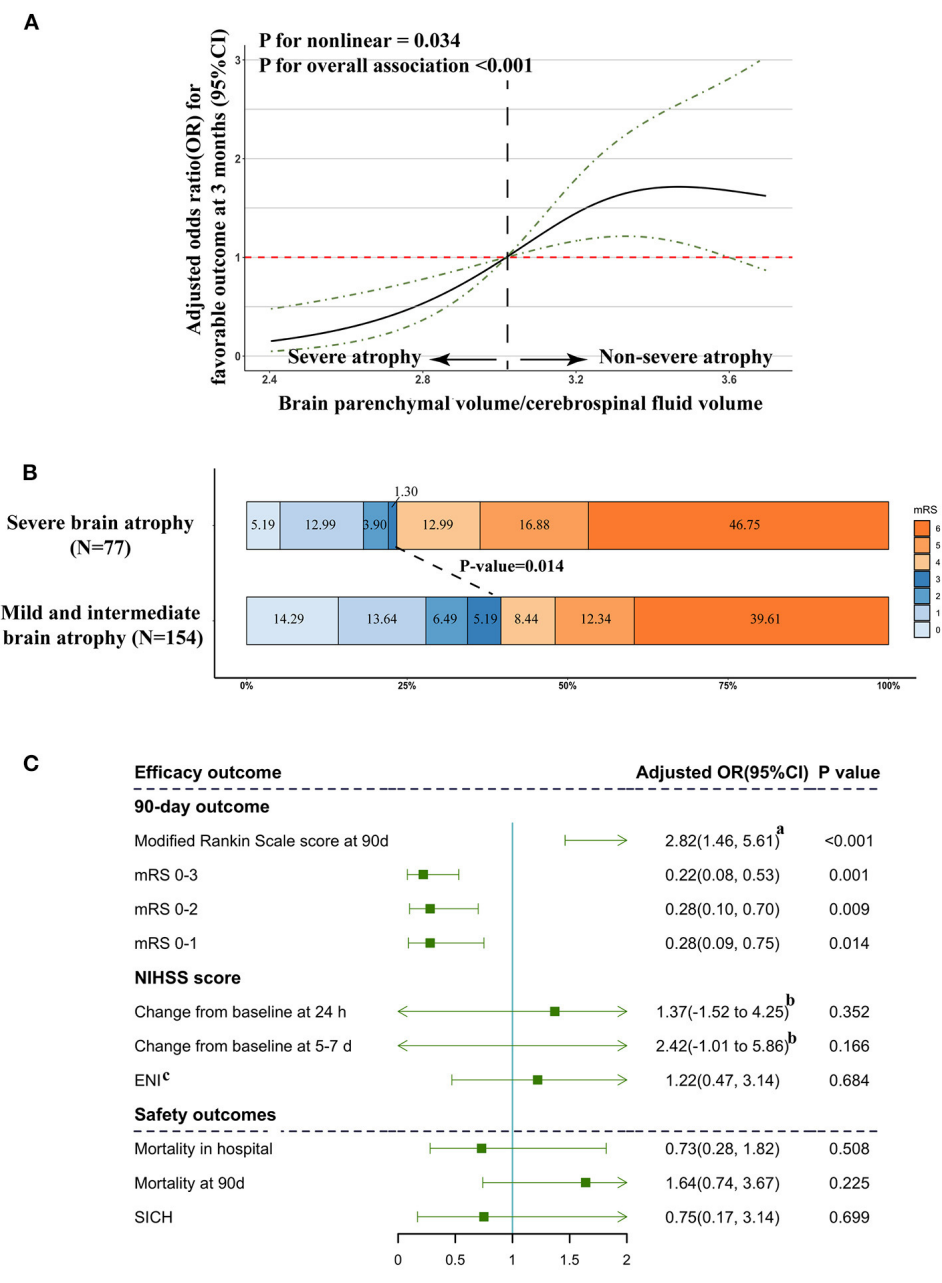
Association of severe brain atrophy with clinical outcome. **(A)** Association of brain atrophy with favorable outcome (mRS ≤ 3) in a restricted cubic spline model. Brain atrophy was estimated by quantifying BPV/CFV. ORs, solid line; 95% CI, dashed lines. **(B)** Primary outcomes according to brain atrophy status. Distribution of modified Rankin Scale (mRS) scores at 3 months in patients treated with endovascular treatment. **(C)** Multivariable logistic regression analysis revealed the relationship between brain atrophy and efficacy outcome and safety outcome. Adjusted estimates of outcome were calculated using multiple regression, taking the following variables into account: age, sex, dyslipidemia, atrial fibrillation, baseline NIHSS score, baseline PC-ASPECTS, mTICI, PC-CS, and onset to recanalization time. (a) Common odds ratio; (b) β and 95% CI values were estimated from a multivariable linear regression model, which was adjusted by multiple regression, taking the following variables into account: age, sex, dyslipidemia, atrial fibrillation, baseline NIHSS score, baseline PC-ASPECTS, mTICI, PC-CS score, and onset to recanalization time. (c) ENI: early neurological improvement was estimated by a reduction of > 8 or return to 0 on NIHSS compared with baseline score at 24 h after EVT.

Our restricted cubic spline analysis detected a significant nonlinear association of brain atrophy levels, with favorable clinical outcomes at 3 months (*P* for non-linear = 0.034, Figure 1A) among patients treated with EVT. With all OR and CI values falling below 1, the unique OR and CI distribution patterns of severe atrophy supported the previous results provided in Table 3 and indicated obviously differential prognostic roles of severe brain atrophy from mild and intermediate atrophy.

The frequency of favorable outcomes (mRS ≤ 3) in patients with severe brain atrophy was significantly lower than that of the other groups (severe vs. non-severe: 23.38 vs. 39.61%, *P* = 0.014; Figure 1B). In the multivariate analysis with adjustment for confounders, the severe brain atrophy level was found to be significantly negatively correlated with the incidence of mRS ≤3 [adjusted OR with 95% CI 0.22 (0.08–0.53), *P* = 0.001, Figure 1C].

The univariate and multivariate analyses were used to explore the association of severe brain atrophy with traditional risk factors in predicting the outcomes for patients with ABAO after EVT (Table 4). Univariate logistic regression showed that severe brain atrophy, NIHSS score at baseline, PC-ASPECTS, and PC-CS were related to the outcomes in patients with ABAO after EVT (all *P* < 0.05). Multivariable logistic regression that included predictors identified by using the univariate analysis (at *P* < 0.05) further identified the following independent predictors of favorable outcomes (mRS ≤ 3) after EVT: severe brain atrophy [adjusted OR with 95% CI, 0.33 (0.15, 0.70), *P* = 0.005], NIHSS score at baseline [adjusted OR with 95% CI, 0.97 (0.93–1.00), *P* = 0.072], PC-ASPECTS [adjusted OR with 95% CI, 1.82 (1.44, 2.36), *P* < 0.001], mTICI [adjusted OR with 95% CI, 1.80 (1.35, 2.50), *P* < 0.001], and PC-CS score [adjusted OR with 95% CI, 1.31 (1.09, 1.60), *P* = 0.005].

**Table 4:** The association of severe brain atrophy and traditional risk factors in predicting outcome.

	**Univariate analysis**		**Multivariate analysis**	
	**OR (95% CI)**	***p*-value**	**Adjusted OR (95% CI)**	***p*-value**
Severe atrophy	0.47 (0.25, 0.85)	0.015	0.33 (0.15, 0.70)	0.005
Age	0.98 (0.96, 1.01)	0.176		
Sex	0.76 (0.41, 1.41)	0.370		
Dyslipidemia	1.55 (0.87, 2.76)	0.134		
Diabetes mellitus	0.79 (0.4, 1.51)	0.484		
Hypertension	1.42 (0.8, 2.58)	0.241		
Atrial fibrillation	1.53 (0.78, 2.98)	0.208		
SBP	1.00 (0.99, 1.02)	0.460		
DBP	1.00 (0.98, 1.01)	0.727		
TIA	0.47 (0.02, 3.27)	0.508		
TOAST	1.11 (0.78, 1.56)	0.555		
Occlusion Sites	0.90 (0.70, 1.15)	0.392		
Thrombolysis treatment	0.81 (0.40, 1.58)	0.551		
Preonset mRS	0.85 (0.39, 1.69)	0.659		
Anesthesia	1.13 (0.81, 1.57)	0.463		
Onset to recanalization time	0.99 (0.99, 1.00)	0.591		
NIHSS baseline	0.94 (0.91, 0.97)	<0.001	0.97 (0.93, 1.00)	0.072
PC-ASPECTS	1.98 (1.59, 2.51)	<0.001	1.82 (1.44, 2.36)	<0.001
mTICI	1.68 (1.33, 2.19)	<0.001	1.80 (1.35, 2.50)	<0.001
PC-CS score	1.89 (1.43, 2.54)	<0.001	1.31 (1.09, 1.60)	0.005

*SBP, systolic blood pressure; DBP, diastolic blood pressure; TIA, transient ischemic attack; TOAST, Trial of ORG 10172 in Acute Stroke Treatment; PC-CS score, posterior circulation collateral system score; mTICI, modified thrombolysis in cerebral infarction; PC-ASPECTS, posterior circulation Alberta Stroke Program Early CT Score*.

### Subgroup Analysis

**Figure 2:**
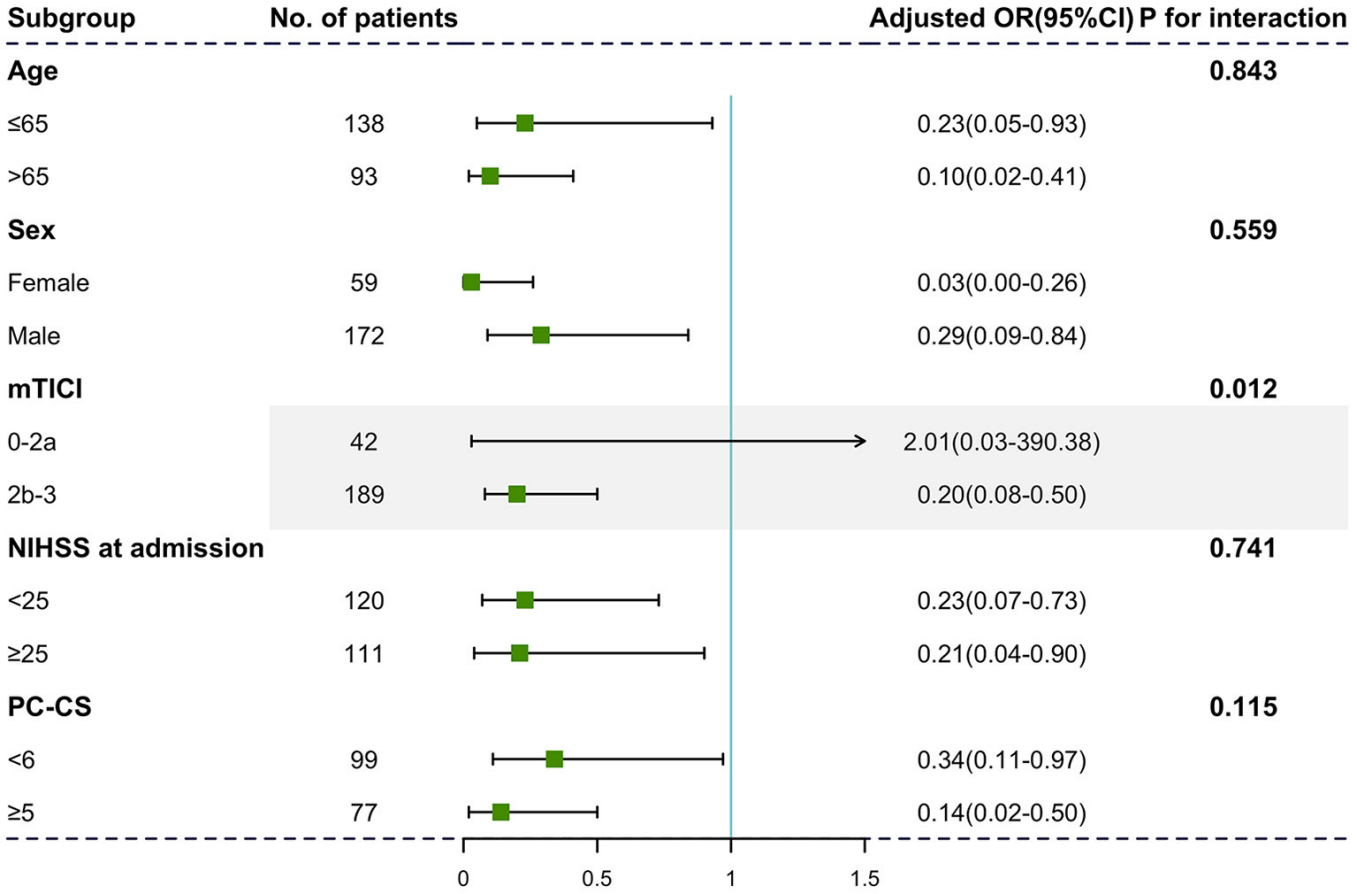
Subgroup analyses of primary outcomes. The forest plot showed the differences in odds ratios for favorable outcomes (defined as the modified Rankin Scale score of 0–3) at 3 months in the prespecified subgroups. Adjusted variables are as follows: age, sex, dyslipidemia, atrial fibrillation, baseline NIHSS score, baseline PC-ASPECTS, mTICI, PC-CS, and onset to recanalization time.

Identical negative effects of severe brain atrophy on 90-day outcome were found in patients in different age strata [Figure 2, age ≤65: adjusted OR with 95% CI, 0.23 (0.05–0.93); age >65: adjusted OR with 95% CI, 0.10 (0.02–0.41)]. Although we did not identify significant interactions between sex and initial NIHSS score, the relationship between severe brain atrophy and unfavorable outcome was more obvious in those with satisfied reperfusion levels (mTICI ≥ 2b, *P* for interaction = 0.012).

### Incremental Effect of the Severe Brain Atrophy Index on the Predictive Value of the Baseline Model

**Figure 3:**
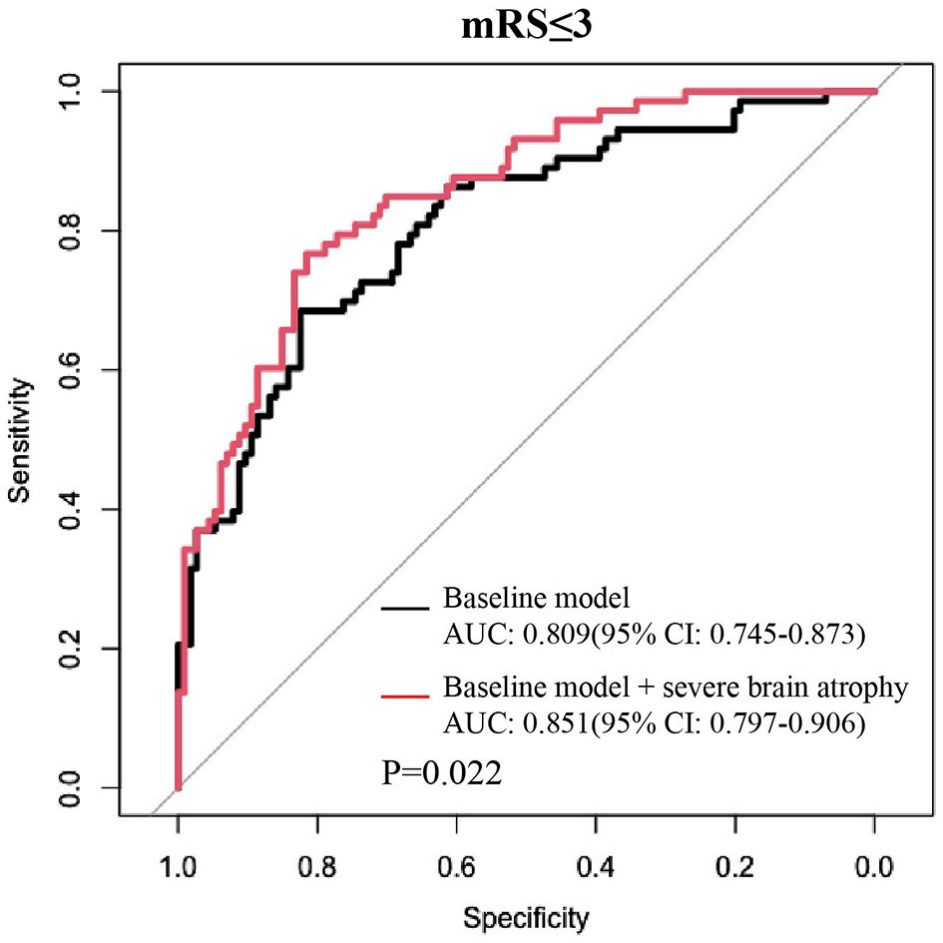
Adding severe brain atrophy index into the baseline model obviously increased its discrimination ability in predicting favorable outcomes among patients with a satisfied reperfusion level (mTICI ≥ 2b). Baseline model: multivariable logistic model constructed by age, sex, dyslipidemia, atrial fibrillation, baseline NIHSS score, baseline PC-ASPECTS, mTICI, PC-CS, and onset to recanalization time.

The addition of the severe brain atrophy index significantly increased the ability of the baseline model to predict the outcomes in individuals with satisfactory reperfusion levels (mTICI ≥ 2b) and yielded a statistically elevated AUC value [baseline model vs. baseline model + severe atrophy: 0.809 (95% CI: 0.75–0.87) vs. 0.851 (95% CI: 0.80–0.91), *P* = 0.022 by using the DeLong's test; Figure 3]. Significant improvements in risk reclassification and discrimination were also detected after adding the severe brain atrophy index into the baseline model, with an NRI of 0.40 (95% CI: 0.12–0.69, *P* = 0.006) and an IDI of 0.04 (95% CI: 0.01–0.07, *P* = 0.005).

The roles of brain atrophy can play in assisting clinical decision-making, namely, receiving medicine or EVT has also been explored. The clinical manifestations of patients receiving medical treatment only are given in Supplementary Table 1, and the outcomes obtained by comparing patients treated with EVT with those treated with medical management only are given in Supplementary Table 2. No differences were observed in favorable outcomes or mortality between patients with ABAO with severe brain atrophy who received different treatment methods (all *P* > 0.05). However, in patients without severe brain atrophy, the EVT cohort presented a remarkably higher rate of favorable functional outcome (39.61 vs. 6.82%; *P* < 0.001) than the medical management only group. As given in Supplementary Table 3, the multivariate analysis confirmed that the following measures were associated with favorable outcome in patients with ABAO without severe brain atrophy: initial NIHSS score [adjusted OR with 95% CI: 0.96 (0.92–0.99), *P* = 0.030], PC-ASPECTS [adjusted OR with 95% CI: 1.78 (1.38–2.36), *P* < 0.001], and intervention treatment [adjusted OR with 95% CI: 11.76 (3.53–55.76), *P* < 0.001]. However, intervention treatment was not significantly related to favorable outcomes in patients with ABAO with severe brain atrophy (*P* > 0.05).

## Discussion

Based on a multi-centered cohort derived from the BASILAR research, this is, to our knowledge, the first study to explore the association between brain atrophy and clinical outcomes for patients with ABAO treated with EVT. Our findings revealed that (1) severe brain atrophy implied poor long-term recovery status, (2) severe brain atrophy could not promote either overall 90-day mortality or in-hospital mortality in patients with ABAO treated with EVT, and (3) adding a severe brain atrophy index to the baseline model yielded a statistically significant improvement in predictions of poor outcomes. The automatic quantitative analysis of brain atrophy applied in this study ensured the reliability of the present analysis and remarkably extended the applicability of our findings.

In the acute stage following EVT, no significant association between brain atrophy and in-hospital mortality was detected in patients with ABAO, a remarkably different result from that of patients with anterior circulation stroke. Candica et al. detected an inverse association between severe atrophy and 7-day death in patients with anterior circulation stroke (Delcourt et al., 2020). Lee et al. demonstrated that brain atrophy might be protective in anterior circulation stroke due to the presence of greater residual intracerebral space available for absorbing the disruption of space-occupying lesions, thus preventing herniation and death (Lee et al., 2010). Two factors might contribute to these discrepancies. First, due to the limited subtentorial space at baseline, severe global brain atrophy might not provide enough room to compensate for the increased regional volume caused by the edema of infratentorial infarctions (Neugebauer et al., 2013). Second, multiple key brain areas such as the midbrain and pons, which regulate a variety of crucial physiological functions, are frequently impaired in patients with ABAO, which might directly result in poor outcomes in the acute phase after EVT, and this dysfunction will not be relieved by the increased residual space available from brain atrophy (Meinel et al., 2019). Overall, the evidence shows that the failure of brain atrophy to improve patient survival guarantees the safety of using severe brain atrophy as a novel prognostic indicator for EVT for ABAO.

As for the long-term prognosis after EVT, this study demonstrated severe cerebral atrophy to be strongly associated with unfavorable 90-day clinical outcomes, which is in line with previous studies on anterior circulation stroke (Pedraza et al., 2020). The decreased ischemic tolerance ability and impaired capacity to adapt and reorganize after stroke might be the dominant mechanisms underlying the prognostic roles of severe brain atrophy in ABAO after EVT (Adduru et al., 2020). Interestingly, we did not detect significant alterations in NIHSS or PC-ASPECTS at admission among different brain atrophy levels. This result agreed with previous findings from the Lauksio, Lee, and Pranita groups focusing on the roles of brain atrophy in outcome evaluation for patients with anterior circulation stroke (Lee et al., 2010; Lauksio et al., 2020; Kaginele et al., 2021). The effect of brain atrophy on prognosis might reflect a reduced ability of recovery after stroke rather than a blunting of the initial stroke severity or PC-ASPECTS (Lee et al., 2010; Schaapsmeerders et al., 2015). In addition, PC-ASPECTS and NIHSS scores were significantly related to onset to treatment time, occlusion sites, and collateral circulation, which might not be affected by brain atrophy status and were also identical among different atrophy groups in this study (Yoshimura et al., 2018; Aoki et al., 2019; Guillaume et al., 2019; Sang et al., 2021). Taken together, the significant relationships that we observed between brain atrophy and favorable outcomes demonstrate the effectiveness of incorporating brain atrophy into the prognosis assessment system for patients with ABAO treated with EVT.

Aging is considered an important contributor to brain atrophy (Moroni et al., 2020). However, the role of aging in determining outcomes in ABAO remains controversial. Kang et al. showed that younger age was significantly associated with a favorable shift in the overall distribution of 90-day mRS (Kang et al., 2018). In contrast, Bouslama et al. found that age was not associated with good outcomes (Bouslama et al., 2017). Our results show that age does not result in significantly greater odds of a poorer clinical outcome after EVT in patients with ABAO, while brain atrophy was a more reliable outcome predictor than age in both univariate and multivariate analyses. Compared with biological aging alone, brain atrophy is the end-organ effect of cumulative risk factors on brain structures that include aging, disease history, education, and vascular risk factors, which might be more directly related to the health status of brain tissues than aging and lead to its outperformance when predicting outcomes (Cole et al., 2015; Pini et al., 2016). These findings proved the necessity of incorporating brain atrophy besides age into the inclusion criteria of future clinical trials on ABAO.

This study had several limitations. First, compared with the CT used in this study, MRI detects cerebral atrophy with better accuracy and provides a more comprehensive imaging assessment of brain region volume. However, CT is the most frequent type of brain image used to diagnose ischemic stroke and has few contraindications. Our approach reflects the clinical practice and generalizes the clinical application of this index. Second, besides atrophy, old infarcts and white matter hyperintensity are also biomarkers for brain frailty (Delcourt et al., 2020). The interaction of old infarcts, white matter hyperintensity, brain atrophy, and clinical outcomes requires further exploration. Third, although we estimated the degree of global brain atrophy automatically and objectively, the atrophy level of specific brain regions such as subtentorial tissues might provide additional prognostic information, and we are carrying out research to further identify imaging markers from specific brain regions.

## Conclusion

Severe brain atrophy might be an independent risk factor for unfavorable clinical outcomes among ABAO subjects after EVT and add prognostic information to the conventional model. Brain atrophy could serve as a novel imaging biomarker to be integrated into the clinical decision-making system to better identify suitable patients for EVT.

## Data Availability Statement

The raw data supporting the conclusions of this article will be made available by the authors, without undue reservation.

## Code Availability Statement

The analyzing codes of this study are available from the corresponding author upon reasonable request.

## Ethics Statement

The studies involving human participants were reviewed and approved by the *post-hoc* analysis of BASILAR registry, which was registered on the Chinese Clinical Trial Registry (ChiCTR1800014759). The study was approved by the research board at each participating center and informed consents were obtained from all patients or their authorized representatives. The patients/participants provided their written informed consent to participate in this study.

## Author Contributions

CL performed most of the experiments, interpreted data, and wrote the first draft of the paper. HL and DW performed a part of experiments and analyzed the data. ZZ, WH, and ZW took part to conceive the study and a part of sample collection. WZ and QY critically edited the manuscript and supervised the study. QY mainly provided funding and designed the study. All authors contributed to the article and approved the submitted version.

## Conflict of Interest

The authors declare that the research was conducted in the absence of any commercial or financial relationships that could be construed as a potential conflict of interest.

## Publisher's Note

All claims expressed in this article are solely those of the authors and do not necessarily represent those of their affiliated organizations, or those of the publisher, the editors and the reviewers. Any product that may be evaluated in this article, or claim that may be made by its manufacturer, is not guaranteed or endorsed by the publisher.
