# Supplementary material for: Severe Brain Atrophy Predicts Poor Clinical Outcome After Endovascular Treatment of Acute Basilar Artery Occlusion: An Automated Volumetric Analysis of a Nationwide Registry
Source: Front Aging Neurosci. 2021 Aug 17;13:720061. doi: 10.3389/fnagi.2021.720061 (PMC8416246; doi:10.3389/fnagi.2021.720061)
Supplement: Supplementary file 1 [file Table_1.DOCX]

Supplemental table 1. Baseline clinical manifestation among ABAO patients stratified by treatment modality and brain atrophy status

|  | Non-severe brain atrophy | | | | Severe brain atrophy | | |
| --- | --- | --- | --- | --- | --- | --- | --- |
|  | SMT  (n=44) | EVT  (n=154) | P | SMT  (n=22) | | EVT  (n=77) | P |
| Age, years, mean±SD | 65.05 ±12.36 | 59.23±10.82 | 0.003 | 75.09±8.64 | | 69.75±11.75 | <0.001 |
| Male,(n%) | 32 (72.73) | 123 (79.87) | 0.420 | 15(68.18) | | 49 (63.64) | <0.001 |
| Baseline NIHSS, median(IQR) | 26.0(16.0-35.0) | 26.0(18.0-32.0) | 0.619 | 29.0(14.0, 34.0) | | 27.0 (16.0-32.0) | 0.813 |
| PC-ASPECTS, median(IQR) | 7.0(6.0- 8.0) | 8.0(7.0-9.0) | 0.021 | 7.50(6.25, 8.00) | | 8.0(6.0-9.0) | 0.715 |
| NIHSS at 24h after EVT, median(IQR) | 32.0(20.75-35.25) | 24.5(12.0-34.0) | 0.007 | 31.0(21.0,35.0) | | 30.0(13.0,35.0) | 0.354 |
| NIHSS at 7days after EVT, median(IQR) | 35.0(27.0-36.0) | 18.0(6.0-35.0) | <0.001 | 31.0(22.0,35.0) | | 23.0(8.0,35.0) | 0.107 |
| Pre-onset mRS (%) |  |  | 0.365 |  | |  | 0.111 |
| 0 | 40 (90.91) | 136(88.31) |  | 18 (81.82) | | 70 (90.91) |  |
| 1 | 2 (4.55) | 15(9.74) |  | 1 (4.55) | | 5 (6.49) |  |
| 2 | 2 (4.55) | 3 (1.95) |  | 3 (13.64) | | 2 (2.60) |  |
| History of risk factors, n(%) | |  |  |  | |  |  |
| Hypertension | 37 (84.09) | 100 (64.94) | 0.025 | 17 (77.27) | | 52 (67.53) | 0.539 |
| Diabetes mellitus | 9 (20.45) | 41 (26.62) | 0.526 | 4(18.18) | | 12(15.58) | 1.000 |
| Dylipidemia | 17 (38.64) | 62 (40.26) | 0.985 | 5 (22.73) | | 11 (14.29) | 0.535 |
| Atrial fibrillation | 5 (11.36) | 22 (14.29) | 0.803 | 4 (18.18) | | 23 (29.87) | 0.416 |
| TIA | 0 (0.00) | 4 (2.60) | 0.637 | 0 (0.00) | | 1 (1.30) | 1.000 |
| TOAST classification, n(%) | |  | 0.383 |  | |  | 0.112 |
| LAA | 31 (70.45) | 109 (70.78) |  | 12 (54.55) | | 42 (54.55) |  |
| CE | 7 (15.91) | 30 (19.48) |  | 5 (22.73) | | 28 (36.36) |  |
| SOE | 0 (0.00) | 4 (2.60) |  | 0 (0.00) | | 2 (2.60) |  |
| SUE | 6 (13.64) | 11 (7.14) |  | 5 (22.73) | | 5 (6.49) |  |
| Imaging factors |  |  |  |  | |  |  |
| Occlusion site, n(%) |  |  | 0.008 |  | |  | 0.035 |
| BA distal | 11(25.00) | 44(28.57) |  | 4(18.18) | | 32(41.59) |  |
| BA middle | 25(56.82) | 47(30.52) |  | 14(63.64) | | 23(29.87) |  |
| BA proximal | 3(6.82) | 29(18.83) |  | 2(9.09) | | 13(16.88) |  |
| V4 | 5(11.36) | 34(22.08) |  | 2(9.09) | | 9(11.69) |  |
| PC-CS score, median(IQR) | 5.50(4.00, 6.25) | 4.00(3.00, 6.00) | 0.075 | 4.00(3.25,6.00) | | 5.00(4.00,6.00) | 0.661 |
| Treatment delay, median(IQR), min | |  |  |  | |  |  |
| Onset to treatment | 270.5(119.75, 467.0) | 325.0(200.75, 512.5) | 0.089 | 205.5(120.25, 480.25) | | 324.0(250.1,440.5) | 0.042 |

^a^mTICI score of 2b or 3 indicates complete recanalization.

Abbreviations: SMT, standard medical therapy; EVT, endovascular treatment; PC-CS score, posterior circulation collateral system score; BA, basilar artery; mTICI, modified thrombolysis in cerebral infarction; V4, V4 segment of vertebral artery; CE, cardioembolism; NIHSS, National Institutes of Health Stroke Scale; pc-ASPECTS, posterior circulation Alberta Stroke Program Early CT Score; SOE, stroke of other determined cause; SUE, stroke of undetermined cause; TOAST, Trial of ORG 10172 in Acute Stroke Treatment.

Supplemental table 2. Clinical outcomes in ABAO cohort stratified by treatment modality and brain atrophy status.

|  | **Non-severe brain atrophy** | |  | **Severe brain atrophy** | | | | |
| --- | --- | --- | --- | --- | --- | --- | --- | --- |
| **Characteristics** | **SMT**  **(n=44)** | **EVT**  **(n=154)** | **P value** | | **SMT**  **(n=22)** | **EVT**  **(n=77)** | | **P value** |
| **Efficacy outcome** |  |  |  | |  |  | |  |
| **90-day outcome** |  |  |  | |  |  | |  |
| mRS 0-3 | 3(6.82) | 61(39.61) | <0.001^a^ | | 2(9.09) | | 18(23.38) | 0.228^a^ |
|  |  |  |  | |  | |  |  |
| mRS 0-2 | 3(6.82) | 53(34.42) | <0.001^a^ | | 2(9.09) | | 17(22.08) | 0.229^a^ |
|  |  |  |  | |  | |  |  |
| mRS 0-1 | 2(4.55) | 43(27.92) | <0.001^a^ | | 2(9.09) | | 14(18.18) | 0.512^a^ |
| **NIHSS score** |  |  |  | |  | |  |  |
| Change from baseline at 24 h, median (IQR) | 0.00[0.00-7.00] | 0.00(-4.00-2.00) | 0.005^b^ | | 0.00[-0.25,7.25] | | 0.00[-2.00-2.00] | 0.291^b^ |
|  |  |  |  | |  | |  |  |
| Change from baseline at 5-7 d, median (IQR) | 2.00[0.00-11.75] | -3.00(-15.25-2.00) | <0.001^b^ | | 1.50[-1.75,15.50] | | 0.00[-10.00-4.00] | 0.058^b^ |
|  |  |  |  | |  | |  |  |
| ENI^c^ | 2(4.55) | 27(17.53) | 0.031^a^ | | 2(9.09) | | 15(19.48) | 0.347^a^ |
| **Safety outcomes** |  |  |  | |  | |  |  |
| Mortality in hospital | 18(37.50) | 30 (19.48) | 0.005^a^ | | 8(36.36) | | 12(15.58) | 0.067^a^ |
|  |  |  |  | |  | |  |  |
| Mortality at 90d | 32(72.73) | 61(39.61) | <0.001^a^ | | 18(81.82) | | 36(46.75) | 0.004^a^ |
|  |  |  |  | |  | |  |  |
| SICH | 0(0) | 7 (4.55) | 0.352^a^ | | 0(0) | | 5(6.49) | 0.584^a^ |

Abbreviations: SMT, standard medical therapy; EVT, endovascular treatment; NIHSS, National Institutes of Health Stroke Scale; mRS: Modified Rankin Scale score at 90 d; SICH, symptomatic intracranial hemorrhage

^a^:Fisher’ exact test.

^b^: Wilcoxon signed rank tests

^c^: ENI: Early neurological improvement was estimated by a reduction of > 8 or return to 0 on NIHSS compared with baseline score at 24 hours after EVT

Supplemental table 3: Predictors of outcome(mRS≤3) in ABAO cohort stratified by treatment modality and brain atrophy status.

|  | Non-severe brain atrophy | | | | Severe brain atrophy | | | |
| --- | --- | --- | --- | --- | --- | --- | --- | --- |
|  | Univariate analysis | | Multivariate analysis | | Univariate analysis | | Multivariate analysis | |
|  | OR(95%CI) | p value | Adjusted OR(95%CI) | p value | OR(95%CI) | p value | Adjusted OR(95%CI) | p value |
| Age | 0.99(0.96-1.02) | 0.469 |  |  | 0.98(0.94-1.02) | 0.232 |  |  |
| Sex | 0.76(0.38-1.56) | 0.440 |  |  | 0.60(0.22-1.66) | 0.315 |  |  |
| Dylipidemia | 1.39(0.76-2.55) | 0.283 |  |  | 0.90(0.19-3.18) | 0.875 |  |  |
| Diabetes mellitus | 0.76(0.37-1.52) | 0.450 |  |  | 2.06(0.58-6.62) | 0.236 |  |  |
| Hypertension | 1.21(0.63-2.36) | 0.572 |  |  | 1.02(0.36-3.16) | 0.974 |  |  |
| Atrial fibrillation | 1.27(0.53-2.93) | 0.574 |  |  | 2.77(0.98-7.79) | 0.051 |  |  |
| TOAST | 1.16(0.90-1.47) | 0.241 |  |  | 0.87(0.52-1.31) | 0.547 |  |  |
| Occlusion Sites | 0.95(0.74-1.20) | 0.670 |  |  | 1.06(0.77-1.40) | 0.673 |  |  |
| Preonset mRS | 0.66(0.26-1.41) | 0.319 |  |  | 0.94(0.26-2.38) | 0.905 |  |  |
| Onset to treatment | 1.00(0.99-1.00) | 0.180 |  |  | 1.00(0.99-1.00) | 0.199 |  |  |
| NIHSS baseline | 0.94(0.91-0.97) | <0.001 | 0.96(0.92-0.99) | 0.030 | 0.93(0.89-0.98) | 0.004 | 0.95(0.89-1.01) | 0.093 |
| PC-ASPECTS | 1.91(1.52-2.48) | <0.001 | 1.78(1.38-2.36) | <0.001 | 3.44(2.11-6.30) | <0.001 | 3.25(1.97-5.98) | <0.001 |
| PC-CS score | 1.20(1.03-1.39) | 0.017 | 1.19(0.99-1.44) | 0.061 | 1.34(1.00-1.83) | 0.053 |  |  |
| Intervention treatment | 8.96(3.08-38.17) | <0.001 | 11.76(3.53-55.76) | <0.001 | 3.05(0.78-20.26) | 0.157 |  |  |

TOAST, Trial of ORG 10172 in Acute Stroke Treatment; PC-CS score, posterior circulation collateral system score; pc-ASPECTS, posterior circulation Alberta Stroke Program Early CT Score; NIHSS, National Institutes of Health Stroke Scale;
